# The Use of Machine Learning to Reduce Overtreatment of the Axilla in Breast Cancer: Retrospective Cohort Study

**DOI:** 10.2196/34600

**Published:** 2022-11-15

**Authors:** Felix Jozsa, Rose Baker, Peter Kelly, Muneer Ahmed, Michael Douek

**Affiliations:** 1 Victor Horsley Department of Neurosurgery National Hospital for Neurology and Neurosurgery London United Kingdom; 2 School of Business University of Salford Salford United Kingdom; 3 Division of Surgery and Interventional Science University College London London United Kingdom; 4 Nuffield Department of Surgical Sciences University of Oxford Oxford United Kingdom

**Keywords:** breast cancer, preoperative screening, machine learning, artificial intelligence, artificial neural network, breast, cancer, axillary node, metastasis, metastatic, preoperative, axillary clearance, metastases, oncology

## Abstract

**Background:**

Patients with early breast cancer undergoing primary surgery, who have low axillary nodal burden, can safely forego axillary node clearance (ANC). However, routine use of axillary ultrasound (AUS) leads to 43% of patients in this group having ANC unnecessarily, following a positive AUS. The intersection of machine learning with medicine can provide innovative ways to understand specific risks within large patient data sets, but this has not yet been trialed in the arena of axillary node management in breast cancer.

**Objective:**

The objective of this study was to assess if machine learning techniques could be used to improve preoperative identification of patients with low and high axillary metastatic burden.

**Methods:**

A single-center retrospective analysis was performed on patients with breast cancer who had a preoperative AUS, and the specificity and sensitivity of AUS were calculated. Standard statistical methods and machine learning methods, including artificial neural network, naive Bayes, support vector machine, and random forest, were applied to the data to see if they could improve the accuracy of preoperative AUS to better discern high and low axillary burden.

**Results:**

The study included 459 patients; 142 (31%) had a positive AUS; among this group, 88 (62%) had 2 or fewer macrometastatic nodes at ANC. Logistic regression outperformed AUS (specificity 0.950 vs 0.809). Of all the methods, the artificial neural network had the highest accuracy (0.919). Interestingly, AUS had the highest sensitivity of all methods (0.777), underlining its utility in this setting.

**Conclusions:**

We demonstrated that machine learning improves identification of the important subgroup of patients with no palpable axillary disease, positive ultrasound, and more than 2 metastatically involved nodes. A negative ultrasound in patients with no palpable lymphadenopathy is highly indicative of low axillary burden, and it is unclear whether sentinel node biopsy adds value in this situation. Further studies with larger patient numbers focusing on specific breast cancer subgroups are required to refine these techniques in this setting.

## Introduction

The contemporary management of the axilla in breast cancer aims to reduce unnecessary intervention while providing optimal oncological safety. Historically, given the well-recognized importance of axillary node status on breast cancer prognosis [1], any patient with axillary disease underwent a complete axillary node clearance (ANC). Several key trials have since reduced the indications for ANC, including evidence that isolated tumor cells [2] and micrometastases [3] were clinically insignificant as well as results of the ACOSOG Z11 trial [4], which demonstrated that in patients with T1-2 breast cancer who had no clinically palpable axillary nodes, with 2 or fewer positive macrometastatically involved axillary nodes at sentinel node biopsy (SNB), no further axillary treatment was necessary. More patients are consequently able to forego ANC, a large surgical procedure with significant morbidity [5], without inferior oncological survival outcomes. The accurate identification of this group of patients is therefore crucially important to ensure they do not receive unnecessary surgical treatment of the axilla.

Axillary ultrasound (AUS) is used nearly ubiquitously in UK breast oncology centers to assess the axilla preoperatively in breast cancer. Typically, a suspicious node viewed on AUS may be biopsied and can be clipped to aid intraoperative identification [6]. When patients are ‘fast-tracked’ to ANC on the basis of a positive AUS, up to 43% of these may have 2 or fewer involved nodes [7] and are thus overtreated. Since AUS was not used in the ACOSOG Z11 trial, this discrepancy remains, and the bypassing of SNB prevents identification of patients who could have safely avoided ANC.

Artificial neural networks are a form of supervised machine learning based on the simplest computational model of a neuron—the ‘perceptron.’ Connections between nodes in consequent layers of a network are weighted probabilistically; following input at the first layer with information about variables describing an item in a data set, which is prelabelled (eg, as ‘dog’ or ‘cat’), the network attempts to correctly categorize the label of the item. This process is repeated on the training set of data while the model updates weights of connections between each iteration to minimize the error of its categorization. Once optimized, it can be deployed on the test set to verify its accuracy.

The aim of this study was to undertake a retrospective pilot study to deploy machine learning methods (ie, artificial neural networks) and traditional statistical models (ie, linear regression) to aid identification of patients with no clinically palpable nodes and a positive preoperative AUS who have low axillary nodal burden. The rational for this is that better identification of this subgroup of patients can reduce the number of patients who undergo unnecessary ANC on the basis of a preoperative positive AUS, which turns out to be clinically insignificant.

## Methods

### Ethics Approval

The study was registered as a clinical audit with the ethics committee of Guy's Hospital, London, United Kingdom and was approved in February 2019 (institutional reference number 7608).

### Data Collection

The first part of this study was to analyze retrospectively the use of preoperative AUS in patients with breast cancer at our tertiary care center. Women with confirmed breast cancer treated at Guy’s Hospital, London, United Kingdom, who had an AUS preoperatively between 2012 and 2014 were retrospectively identified from a departmental database. The results of the AUS and the patients’ sex; age; date of birth; primary tumor size, grade, and type; as well as receptor phenotype were recorded alongside the results of any axillary surgical intervention and breast surgery. Lymph nodes were evaluated with ultrasound using the following criteria for reporting an abnormal node: diffuse or focal cortical enlargement, loss of lymph node fatty hilum, and enlarged nodal size [8]. All data were fully anonymized.

The second part of this study was to use machine learning and statistical methods to try and improve identification of patients with high or low axillary burden. High burden in patients was defined as more than 2 macrometastatic axillary nodes. Low burden was defined as 0, 1, or 2 macrometastatic nodes or isolated tumor cells or micrometastases in patients.

Both types of models were given the following patient characteristics to predict nodal burden: patient age, estrogen receptor and HER2 status, tumor grade, presence of associated ductal carcinoma in-situ, tumor type (eg, invasive ductal carcinoma and invasive lobular carcinoma), tumor size, presence of lymphovascular invasion, and the result of a preoperative AUS.

### Machine Learning Methods

After collection and deidentification of data, the data set was preprocessed using pandas [9], matplotlib [10], and scikit [11], which are open-source data analysis and manipulation tools built in the Python programming language. A total of 70% of the data was randomly selected to form the training set, on which predictive models were developed, with the other 30% designated as the test set. The resultant nodal burden of each patient was labelled as 1 or 0 to indicate low and high nodal burden respectively, and this feature was designated as the label to be predicted by the model. Categorical variables were one-hot encoded, and numerical variables were scaled to between 0 and 1 using the MinMaxScaler function. TensorFlow [12] and Keras were used to design the artificial neural network (ANN). A dense, feed-forward ANN with 3 layers of 11, 6, and 1 neuron, respectively, was constructed with backpropagation optimized using Adam [13]. Support vector machine, random forest, and naive Bayes classifier methods were also used for comparison with the ANN.

### Statistical Methods

Logistic regression is a well-known and widely used technique for predicting binary variables and carrying out discriminant analysis when the predictor variables are not all normally distributed [14]. It was used for classification here by choosing the predicted group as the group with the larger predicted probability of membership.

Logistic regression is a standard methodology, and the only nontrivial problem was estimation of the sensitivity and specificity. These would have been overestimated if computed in-sample from fitted data. We therefore used a computationally feasible method for out-of-sample estimation—k-fold cross-validation; this is a better use of data compared to estimating sensitivity on a hold-out sample.

The model was fitted k times, leaving out each ‘fold’ in turn, and predictions were then made for that fold using the fit to the other folds only. Folds were produced by shuffling high and low burden cases separately and then dividing the sample so that the percentage of high-burden cases was as equal between the folds as possible. We used 5 folds, which is usually taken as sufficient, and moving to 10 folds made very little difference.

The method is not Bayesian but can be made so using a ‘vague prior.’ Laplace’s method of integration was used to obtain a Bayesian solution, and when this was done, the probability that a patient had low or high burden shifted slightly toward 1/2, by about 0.02, so the Bayesian methodology gave a slightly less certain prediction. However, the classification was unchanged, so the Bayesian refinement was not used.

## Results

A total of 459 patients with breast cancer who had undergone a preoperative AUS before SNB or primary surgery with ANC were included. Patient characteristics are detailed in Table 1. All patients were women, with a mean age of 57.1 (SD 13.9) years. Mean tumor size was 28.3 (SD 24.05) mm, of which 319 (69.5%) were invasive ductal carcinoma, and 69 (15%) were invasive lobular carcinoma.

**Table 1:** Patient characteristics. All patients were female.

Characteristics			All patients (N=459)		Low burden (≤2 nodes; n=392)		High burden (>2 nodes; n=67)
Age (years), mean (range, SD)			57.11 (28-88, 13.85)		57.48 (29-88, 13.80)		54.97 (28-86, 14.05)
Tumor size (mm), mean (range, SD)			28.29 (1.1-180, 24.05)		25.48 (1.1-180, 20.4)		44.99 (3-180, 35.1)
**Tumor histology, n (%)**							
	Invasive ductal carcinoma	319 (69.5)		260 (66.3)		55 (82.1)	
	Invasive lobular carcinoma	69 (15)		56 (14.3)		8 (11.9)	
	Other invasive types	41 (8.9)		39 (10)		2 (3)	
	Isolated in situ disease	30 (6.5)		30 (7.7)		0 (0)	
**Tumor grade, n (%)**							
	1	48 (10.5)		45 (11.5)		3 (4.5)	
	2	204 (44.4)		177 (45.2)		27 (40.3)	
	3	176 (38.3)		139 (35.5)		37 (55.2)	
	Not specified	2 (0.4)		2 (0.5)		0 (0)	
**Invasive tumor with associated DCIS^a^, n/N (%)^b^**							
	High grade	194/269 (72.1)		7/222 (3.2)		0/47 (0)	
	Intermediate grade	68/269 (25.3)		58/222 (26.1)		10/47 (21.3)	
	Low grade	7/269 (2.6)		157/222 (70.7)		37/47 (78.7)	
**Receptor phenotype, n (%)**							
	Luminal A	332 (72.3)		283 (72.2)		67 (71.6)	
	Luminal B	30 (6.5)		23 (5.9)		48 (10.5)	
	Triple negative	65 (14.2)		58 (14.8)		6 (9)	
	HER2	13 (2.8)		8 (2.2)		5 (7.5)	
	Not specified	19 (14.1)		20 (5.1)		1 (1.5)	
**Primary surgery, n (%)**							
	WLE^c^	257 (56)		210 (53.6)		25 (37.3)	
	Mastectomy	193 (42)		151 (38.5)		41 (61.2)	
	Lymphovascular invasion present	114 (24.8)		73 (18.6)		41 (61.2)	

^a^DCIS: ductal carcinoma in situ.

^b^The total number of patients in this category was 269/459 (58.6%); the total number of patients with low burden (≤2 nodes) was 222 (56.6%); and the total number of patients with high burden (>2 nodes) was 47 (70.2%). All the other percentages under this category are calculated based on these denominators.

^c^WLE: wide local excision.

### Accuracy of Preoperative AUS

The preoperative AUS was positive in 142 (31%), negative in 285 (62.09%), and inconclusive in 32 (6.97%) patients. Among patients with a positive ultrasound, 54 (38.03%) had more than 2 positive axillary nodes at ANC, and 88 (62%) had 2 or fewer nodes. Among patients with a negative ultrasound, 304 (95.9%) had 2 or fewer than 2 positive nodes at SNB (Table 2). In the subgroup of patients with a negative AUS and a tumor size of 20 mm or less, the number of patients with 2 or fewer positive nodes at SNB was 5 (2.78%). The sensitivity and specificity of ultrasound overall from these data was 0.809 (95% CI 0.715-0.902) and 0.777 (95% CI 0.736-0.818), respectively. The accuracy was 0.820 (95% CI 0.778-0.862).

**Table 2:** Axillary nodal burden of patients with positive and negative ultrasound.

Nodal burden	Ultrasound negative (N=317), n (%)	Ultrasound positive (N=142), n (%)
Two or fewer nodes	304 (95.9)	88 (62)
More than 2 nodes	13 (4.1)	54 (38)

### Application of Machine Learning and Statistical Models

All machine learning and statistical models applied to these data delivered improved specificity when compared to preoperative AUS (Table 3).

The best performing model was logistic regression, with a specificity of 0.950. This was achieved by sacrificing sensitivity, which was 0.462. If logistic regression had been used on this patient cohort, 66/459 (14.3%) patients who had a positive AUS and low axillary burden would have been identified as such and avoided unnecessary ANC; 20/459 (4.3%) patients would have been wrongly classified as having low burden, but these would then have undergone SNB as per current practice and likely been identified as having high burden at that point. The most important covariates identified by logistic regression were abnormal AUS, lymphovascular invasion, tumor size, as well as invasive ductal and invasive lobular carcinoma tumor types.

**Figure 1:**
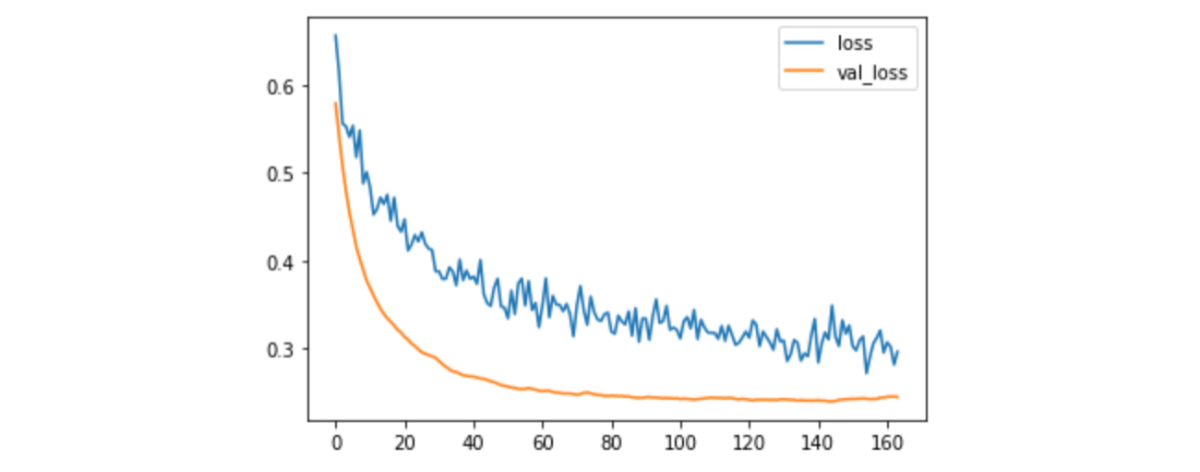
Training of the artificial neural network over 163 epochs.

The ANN, support vector machine, naive Bayes, and random forest classifiers all outperformed preoperative ultrasound’s specificity, but none were able to improve on its sensitivity (Table 3). The ANN was stopped early after 163 epochs of training (Figure 1), reaching a specificity of 0.9355 and a sensitivity of 0.7273. As such, the ANN had the highest accuracy (0.919) of all models, including logistic regression. When performing on the test set, the ANN correctly identified 21 of the 24 patients with a positive ultrasound and low burden.

**Table 3:** Comparison of preoperative ultrasound with logistic regression and machine learning models.

Method	Specificity	Sensitivity	Accuracy
Preoperative axillary ultrasound	0.809	0.777	0.820
Logistic regression	0.950	0.462	0.880
Naive Bayes	0.947	0.476	0.874
Artificial neural network	0.936	0.727	0.919
Support vector machine	0.934	0.615	0.904
Random forest	0.911	0.455	0.874

## Discussion

### Principal Findings

Our results demonstrate that logistic regression and machine learning methods can be used effectively to reduce the number of patients undergoing ANC unnecessarily. As current practice leads to 43% of patients with early breast cancer, nonpalpable axillary nodes, and a positive ultrasound receiving such overtreatment, this is a valuable addition to the preoperative workup of breast cancer patients, and there are significant implications on clinical practice.

In this data set, logistic regression performed best. The particular success of logistic regression’s high specificity came at a cost of poor sensitivity. However, this trade-off is favorable in the case of axillary staging because patients deemed as low risk will undergo SNB. Thus, the potential group of patients wrongly classified as having low burden by logistic regression will be identified and not left without treatment. For this reason, despite the ANN’s accuracy outperforming the other models, logistic regression is the best model for the problem presented by the data. Indeed**,** a recent meta-analysis of clinical prediction models found that logistic regression tends to perform better than machine learning methods in this setting [15] as a predictor of disease in a data set of relatively low dimensions and size.

This study confirms that machine learning can be successfully deployed in the preoperative assessment of patients with breast cancer, despite not being able to outperform logistic regression’s optimization of specificity for this task. The ANN developed the greatest overall accuracy, meaning it would have been the most useful tool if SNB following negative imaging was not standard of care. Larger and higher dimensional data sets will likely provide an arena in which machine learning can excel, particularly when considering its potential to combine image analysis techniques using convolutional neural networks and standard data in the form used in this study [16].

The fact that none of the models could improve on the sensitivity of AUS underlines the value of this imaging modality for helping rule out axillary disease in the clinically node negative breast cancer population. Evidence from a meta-analysis of 5139 patients showed that ultrasound’s negative predictive value was 0.951 (95% CI 0.941-0.960) in this setting [17]. Despite this, patients with a negative ultrasound still undergo a SNB, and this may be considered surgical overtreatment in the same sense that ANC is used unnecessarily in the ultrasound positive group. This issue is currently being addressed in the SOUND randomized control trial [18]. Adaption of machine learning and statistical methods could be used on large data sets to help identify the approximately 4% of patients with no clinically palpable disease and a negative ultrasound but with more than 2 macrometastatically involved axillary nodes. This could lead to future selective use of SNB in this patient subgroup, analogous to the selective use of ANC, which is now common practice among patients with nodal burden identified on SNB.

There are several limitations to this study. They stem principally from the fact that this study is a proof-of-concept idea demonstrating the application of machine learning techniques in a breast surgery cohort, applied to a specific clinical and radiological problem within the general breast cancer patient population but not able to further delineate important risk differences between subgroups in this population. For example, it has not included several important patient factors and data points, which may prove important to refining models before implementation in a real-world scenario; examples of parameters that the authors would like to include in further models include menopausal status and lymph node biopsy pathology results. A further limitation of this study’s applicability to clinical practice was that it did not consider patients undergoing primary systemic therapy, the indications for which have increased [19]. In this patient group, the use of ultrasound is less important as staging magnetic resonance imaging is often used alongside SNB to assess response to treatment. Another key limitation of this study was that our data set was relatively small; deployment of the same models on much larger sets of patient data would be necessary to further validate our results. Furthermore, with larger training sets, model performance may improve. This could allow for suture large studies on specific breast cancer patient subgroups, for example invasive lobular carcinoma. A further interesting future consideration will be to include particular aspects of ultrasound data, for example cortex to hilum ratios when computing predictive models, or to combine data predictive methods with computer vision techniques looking directly at the ultrasound images obtained from each patient.

### Conclusions

AUS’s poor specificity renders it ineffective to reliably identify patients with a clinically negative axilla and significant nodal burden (ie, more than 2 macrometastatic nodes), despite it being attractive as a noninvasive and widely available tool. The addition of logistic regression and machine learning methods can provide valuable predictions based on patient characteristics and the AUS result, which can greatly reduce the surgical overtreatment of the axilla and significantly improve the accuracy of identification of high nodal burden among patients with no clinically palpable disease. This two-part improvement in preoperative axillary staging is highly desirable and has the potential to spare many patients unnecessary axillary surgery; however, given the heterogenous nature of the patient population in this study, further refinement of the models with international multicenter trials are warranted to confirm the results.

## Abbreviations

ANC: axillary node clearance
ANN: artificial neural network
AUS: axillary ultrasound
SNB: sentinel node biopsy
